# An Integrated Strategy to Identify and Quantify the Quality Markers of Xinkeshu Tablets Based on Spectrum-Effect Relationship, Network Pharmacology, Plasma Pharmacochemistry, and Pharmacodynamics of Zebrafish

**DOI:** 10.3389/fphar.2022.899038

**Published:** 2022-05-23

**Authors:** Yongheng Wei, Lei Nie, Lele Gao, Liang Zhong, Zhongyu Sun, Xiangchun Yang, Jianan Yue, Yingzi Zeng, Lian Li, Jing Sun, Hengchang Zang

**Affiliations:** ^1^ NMPA Key Laboratory for Technology Research and Evaluation of Drug Products, School of Pharmaceutical Sciences, Cheeloo College of Medicine, Shandong University, Jinan, China; ^2^ Shandong Wohua Pharmaceutical Technology Co., Ltd., Weifang, China; ^3^ Key Laboratory of Chemical Biology (Ministry of Education), Shandong University, Jinan, China; ^4^ Qinghai Provincial Key Laboratory of Qinghai-Tibet Plateau Biological Resources, Northwest Institute of Plateau Biology, Chinese Academy of Sciences, Xining, China; ^5^ National Glycoengineering Research Center, Shandong University, Jinan, China

**Keywords:** quality markers, Xinkeshu tablets, spectrum-effect relationship, network pharmacology, plasma pharmacochemistry, pharmacodynamics of zebrafish

## Abstract

Xinkeshu tablets (XKST), a traditional Chinese patent medicine (CPM), have served in the clinical treatment of cardiovascular diseases (CVDs) for decades. However, its pharmacodyamic material basis was still unclear, and the holistic quality control has not been well established due to the lack of systematic research on the quality markers. In this experiment, the heart rate recovery rate of a zebrafish larva was used to evaluate the traditional pharmacological effect of XKST i.e., antiarrhythmic effect. The HPLC fingerprints of 16 batches of XKST samples were obtained, and antiarrhythmic components of XKST were identified by establishing the spectrum-effect relationship between HPLC fingerprints and heart rate recovery rate of zebrafish larva with orthogonal signal correction and partial least squares regression (OSC–PLSR) analysis. The anticardiovascular disease components of XKST were identified by mapping the targets related to CVDs in network pharmacology. The compounds of XKST absorbed and exposed *in vivo* were identified by ultra-high performance liquid chromatography Q-Exactive high-resolution mass spectrometry (UHPLC-Q-Exactive HRMS). Based on the earlier studies, combined with five principles for identifying quality markers and verified by a zebrafish arrhythmia model, danshensu, salvianolic acid A, salvianolic acid B, daidzein, and puerarin were identified as quality markers of XKST. In total, 16 batches of XKST samples were further quantified with the method established in this study. Our study laid the foundation for the quality control of XKST. The integrated strategy used in the study of XKST could be applied for the identification and quantification of quality markers of other CPMs as well.

## 1 Introduction

Traditional Chinese medicine (TCM) has played a major role in China’s national health care system due to its robust security and good clinical efficacy and has raised significant interests in the international medical field (Zhang et al., 2019). The chemical composition of TCM, especially for Chinese patent medicine (CPM) that is constituted by several herbs, is complex and has various action targets, while the quality control standards of most preparations are simply in accordance with the index components in *Chinese Pharmacopoeia*, which is still not perfect enough to meet the requirements of effectiveness, stability, and controllability (Lau et al., 2019). Considering the characteristics of the Chinese medicine system including biological properties, manufacturing process, and compatibility theory, academician Liu proposed the new concept of quality markers in 2016 to improve the quality and quality control of TCM (Yang et al., 2017). The screening of quality markers should conform to “five principles,” that is, delivery and traceability, specificity, compatibility, effectiveness, and measurability. Until now, multidisciplinary techniques, integrating natural product chemistry, analytical chemistry, bionics, chemometrics, pharmacology, systems biology, and pharmacodynamics have been developed to serve as paradigms for the identification and determination of quality markers (Yang et al., 2017; Liang et al., 2021).

Xinkeshu tablets (XKST), a traditional Chinese patent medicine (CPM), which consist of five herbs, *Salvia miltiorrhiza* Bunge (danshen in Chinese), *Pueraria montana var. lobata* (Willd.) Maesen & S.M.Almeida ex Sanjappa & Predeep (Gegen in Chinese), *Crataegus pinnatifida* Bunge (Shanzha in Chinese), *Panax notoginseng* (Burkill) F.H.Chen (Sanqi in Chinese), and *Aucklandia costus* Falc (Muxiang in Chinese), in a ratio of 15:15:15:1:1, have been extensively used for the treatment of cardiovascular diseases (CVDs) in China for decades (Peng et al., 2011). Pharmacological studies have revealed that XKST can effectively regulate the abnormal changes in blood lipid and lipid peroxide and have a protective effect on myocardial ischemia and reperfusion injury in animal models (Liu et al., 2014a; Liu et al., 2014b; Yang et al., 2018). However, up to now, the quality-control index of XKST is only limited to a few index ingredients in Chinese Pharmacopoeia, which cannot reflect the characteristics of overall pharmacological effects through multi-components of TCM prescription, and there is only little literature available for the holistic quality identification of XKST (Peng et al., 2011; Wang et al., 2012; Qing et al., 2022). As a consequence, it is urgent to provide an adequate quality control procedure based on the quality markers for revealing and controlling the comprehensive quality of XKST.

The cardiovascular systems of the zebrafish are anatomically and physiologically similar to those of mammals. Zebrafish share a very high genetic similarity with humans, about 87% (Seto et al., 2015). Moreover, zebrafish have many biological advantages, such as high transparency, high fertility, high flux, low-cost, short life cycle, easy to maintain, and fewer test compounds required (Shi et al., 2020). At present, zebrafish have become an attractive model for studying the pathogenesis and pharmacological activities of drugs for CVDs due to its unique characteristics and availability of advanced genetic techniques (Seto et al., 2015). Arrhythmia, as an important group of CVDs, is the irregular rhythm of the heart which occurs when electrical impulses of the normal heart beats are disrupted (Mandal et al., 2021). It may occur alone or in association with other CVDs. In view of this, we evaluated the pharmacological effects of XKST against CVDs using the zebrafish arrhythmia model.

**FIGURE 1:**
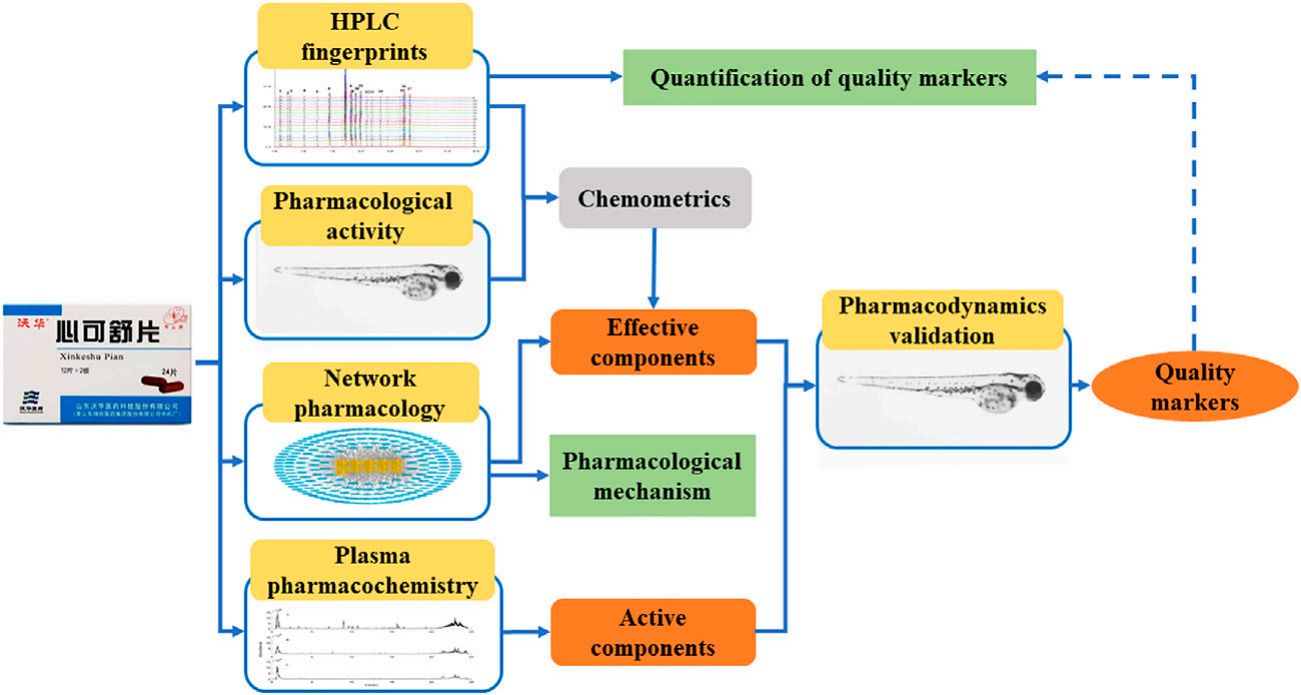
Strategy to identify and quantify quality markers of XKST.

Based on the concept and theories of quality markers, this study proposed the strategy, integrated with spectrum-effect relationship, network pharmacology, plasma pharmacochemistry, and pharmacodynamics of zebrafish, to identify and quantify the quality markers of XKST as shown in Figure 1. The effective components were found on the basis of studying spectrum-effect relationship and network pharmacology. At the same time, the mechanism of action of the medicine was explained by network pharmacology. Active components which were absorbed and exposed *in vivo* were identified based on plasma pharmacochemistry. Based on the effective components and active components earlier identified, combined with five principles of quality markers determination, the quality markers of XKST were identified after being verified by a zebrafish arrhythmia model. The identified quality markers were quantified using the HPLC method established in the study finally. This study would provide an experimental basis to achieve a comprehensive and reliable quality control evaluation of XKST and may provide a perspective for the exploration of quality markers of CPM.

## 2 Experimental

### 2.1 Instrumentation

The HPLC fingerprint analysis was conducted on an Agilent 1260 HPLC system using an ultraviolet detector (UVD). A thermo LC–MS system equipped with ultimate 3000 UHPLC (Thermo Fisher Scientific Inc., MA, United States) coupled with hybrid quadrupole-orbit trap mass spectrometry (Thermo LC/MS Division, CA, United States) was used. The type of high speed refrigerated centrifuge was TGL-16gR (Shanghai Anting Scientific Instrument Factory, Shanghai, China), the type of the termovap sample concentrator was DCY-24S (Qingdao Haike Instrument Co., Ltd., Qingdao, China), the type of the microplate reader was Synergy/H1 (BioTek Instruments, Inc., United States), and the type of stereomicroscope was OLYMPUS SZX7 (Olympus corporation, Tokyo, Japan).

### 2.2 Reagents and Materials

In total, 16 batches of XKST were produced by Shandong Wohua Pharmaceutical Co., Ltd., and their batch numbers were 0181046 (S1), 0181248 (S2), 0181268 (S3), 0190146 (S4), 0190147 (S5), 0190148 (S6), 0190149 (S7), 0190150 (S8), 0190252 (S9), 0190253 (S10), 0190254 (S11), 0190256 (S12), 0190257 (S13), 0190258 (S14), 0190259 (S15), and 0190260 (S16). Reference compounds including danshensu (CAS 76822-21-4, Lot D03611806004), protocatechualdehyde (CAS 139-85-5, Lot Y-032-170426), daidzin (CAS 552-66-9, Lot D-013-181216), daidzein (CAS 486-66-8, Lot D01611812016), 3′-hydroxypuerarin (CAS 117060-54-5, Lot Q0170201011), puerarin (CAS 3681-99-0, Lot G00811804026), salvianolic acid A (CAS 96574-01-5, Lot D01102104026), and salvianolic acid B(CAS 121521-90-2, Lot D01202110027) were purchased from Chengdu Ruifensi Biotechnology Co., Ltd., and salvianolic acid D (CAS #142998-47-8, Lot J24GB152510), 3′-methoxypuerarin (CAS #117047-07-1, Lot P07N9F73425), ononin (CAS #486-62-4, Lot B20214), quinic acid (CAS #77-95-2, Lot B21879), puerarin (CAS #3681-99-0, Lot S02M9854875), biochanin A (CAS #491-80-5, Lot B21589), and terfenadine (CAS #50679-08-8, Lot M27D10L106851) were purchased from Shanghai Yuanye Biotechnology Co., Ltd. All the chemicals were with a purity of greater than 98%. Pronase E (CAS 9036-06-0, Lot P8360) and isoprenaline hydrochloride (ISO, CAS 51-30-9, Lot 720B024) were purchased from Beijing Suolaibao Technology Co., Ltd. Dimethyl sulfoxide (DMSO) was purchased from Sangon Biotech (Shanghai) Co., Ltd. Acetonitrile (HPLC grade), phosphate (HPLC grade), and formic acid (HPLC grade) were purchased from Tianjin Kemiou Chemical Reagent Co., Ltd., and methanol (analysis grade) was purchased from Sinopharm Chemical Reagent Co., Ltd. Double distilled water for at least 18.2MΩ was purified by using an ultrapure water system produced by MILLIPLEX Company in France.

### 2.3 HPLC Fingerprints of XKST

#### 2.3.1 Chromatographic Conditions

In previous work, our research group had developed and verified the determination method of XKST fingerprint (Wang et al., 2012). On the basis of the original method, we improved it and made a method validation. The HPLC fingerprint analysis was conducted on an Agilent HPLC system using an ultraviolet detector (UVD) and Agilent InfinityLab ROZE C18 column (2.1 × 100 mm, 2.7 μm) maintained at 30°C. The mobile phases were 0.05% phosphoric acid in deionized water (A) and acetonitrile (B) (v/v), and a flow rate of 0.4 ml/min was utilized. The gradient elution program was as follows: 0–7 min, 8%–12% B; 7–10 min, 12%–17% B; 10–18 min, 17%–38% B; 18–22 min, 38%–90% B; and 22–25 min, 90%–10% B with a linear gradient. An injection volume of 2 μl was used for each run. The detection wavelength was set at 278 nm for the establishment of fingerprints.

#### 2.3.2 Sample Preparation

Sixteen batches of XKST were finely ground and accurately weighed 0.1 g, transferred into 5-ml volumetric flasks, and then these samples were dissolved in 70% methanol and diluted to 5 ml. After ultrasonic treatment for 20 min, centrifugation was performed at 9,000 r/min for 10 min. The supernatant filtered with a 0.22 μm filter membrane was considered as the test solution for HPLC analysis and UHPLC-Q-Exactive HRMS analysis. For the sample preparation, 1 mg of danshensu sodium, protocatechualdehyde, 3′-hydroxypuerarin, puerarin, 3′-methoxypuerarin, daidzein-7-glucoside, daidzein, salvianolic acid B, salvianolic acid A, salvianolic acid D, ononin, quinic acid, puerarin, and biochanin A were accurately weighed and transferred into 10-ml volumetric flasks. These reference samples were solved with 70% methanol and diluted to 10 ml, respectively, then filtered with a 0.22 μm filter membrane as reference solutions for HPLC analysis and UHPLC-Q-Exactive HRMS analysis. Sixteen batches of fine powder of XKST were weighed 1.0g, and solved with 70% methanol and diluted to 10 ml. Following ultrasonic treatment for 60 min and filtration, the filtrate was then placed into an evaporating dish and dried in a water bath at 70°C for 2 h, and then dried for 70 h in a vacuum oven at 70°C to obtain the dry extract. The dry extract was dissolved in 50% DMSO solution (DMSO and water in equal proportion) at the concentration of 200 mg/ml in terms of the original medicine as a test solution for the zebrafish pharmacodynamics experiment. The reference samples were solved with 50% DMSO solution in different corresponding doses as a reference solution for the zebrafish pharmacodynamics experiment.

### 2.4 Arrhythmia Assays in Zebrafish

AB line zebrafish (provided by the Engineering Research Center of Zebrafish Models for Human Diseases and Drug Screening of Shandong Province) were kept under a 14 h/10 h light/dark cycle at a constant temperature (28 ± 0.5°C) in a breeding system (Shanghai Haisheng Biotech Co., Ltd.). Brine shrimp were provided twice daily at 9:00 and 16:00. Adult male and female zebrafish in a 1:1 ratio were placed on opposite sides of a divider in a breeding tank the night before fertilization, and the next morning, the zebrafish laid eggs by natural mating soon after first light. The embryos were collected within 30 min after spawning and rinsed three times with fresh water. The clean embryos were moved to tanks with fish water (5 mM NaCl, 0.17 mM KCl, 0.4 mM CaCl_2_, and 0.16 mM MgSO_4_) and cultured at 28.5°C for subsequent experiments. The zebrafish assay was approved by the Ethics Committee of the Biology Institute of Shandong Academy of Science. All experiments followed the protocols outlined by the Engineering Research Center of Zebrafish Models for Human Diseases and Drug Screening of Shandong Province. In 24 h post-fertilization (hpf), fish larvae were picked and membranes of these larvae were ruptured artificially using 1 mg/ml pronase E. Larvae were distributed into a 24-well plate and 10–12 larvae in each well with system fish water for treatment. The control group (Con) was treated with 0.5% DMSO. The test groups were treated with test samples at the indicated concentrations (as shown in the result) and terfenadine at the concentration of 7.5 µM. The model group was treated with terfenadine at the concentration of 7.5 µM. The positive group was treated with ISO at the concentration of 20 µM and terfenadine at the concentration of 7.5 µM. Each well was filled to 2 ml with fish water medium and incubated at 28°C in a light incubator controlled by light and temperature. In 48 hpf, the beating of the zebrafish larvae heart was recorded under a stereomicroscope. The heart rate recovery rate (*R*_*i*_) of the components was calculated using the following formula:

$$Ri=\frac{B_{i}-B_{M}}{B_{C}-B_{M}}\;\times \;100\%,$$

where *R*_*i*_ is the normalized heart rate recovery rate of the test solution *i*, *B*_*i*_ is the beats of larvae treated by test solution *i*, *B*_*M*_ is the beats of larvae treated by terfenadine, and *B*_*C*_ is the beats of larvae treated in the control group.

### 2.5 Network Pharmacology

#### 2.5.1 Determination of the Chemical Composition and Target of XKST

A total of 62 compounds in XKST were identified in combination with our previous HPLC–MS/MS component identification studies on XKST and related literature reports (Peng et al., 2011; Wang et al., 2012; Qing et al., 2022), and the targets of these compounds were predicted through the Swiss Target Prediction database (http://www.swisstargetprediction.ch/), PubChem database (https://pubchem.ncbi.nlm.nih.gov/), and STITCH database (http://stitch.embl.de/).

#### 2.5.2 Identification of Disease Targets

Disease targets associated with CVDs were gathered through Online Mendelian Inheritance in Man (OMIM) database (http://www.omim.org/), DrugBank database (https://www.drugbank.ca/), Pharmacogenetics and Pharmcogenomics Knowledge Base (PharmGKB) database (http://www.omim.org/) and Kyoto Encyclopedia of Genes and Genomes (KEGG) database (http://www.kegg.jp/).

#### 2.5.3 Construction of Compound–Target Network

XKST targets and disease targets were mapped to find compounds acting on disease targets related to CVDs, which can be potentially active components. The targets related to CVDs of the compounds were uploaded to Cytoscape 3.2.1 software to generate a network reflecting the interaction between the compounds and the targets, so as to explore the pharmacological mechanism of XKST.

#### 2.5.4 Enrichment Analysis of the Kyoto Encyclopedia of Genes and Genomes

The enrichment analysis of the KEGG pathway was performed on the target using the ClueGO plugin to explain the role of the target of the compound in the signal pathway. The principal role of XKST in the treatment of CVDs was screened by *p* < 0.05, and the enrichment analysis results were visualized by R software (http://www.ehbio.com/ImageGP/).

### 2.6 Analysis of Components in Plasma

#### 2.6.1 Rats Handling

In total, 12 male Wistar rats (230 ± 20 g) used in this study were obtained from the Experimental Animal Center of Shandong University (Shandong, China, approval no. SYXK(Lu)2013-0001). The rats were allowed to acclimatize to the standard conditions for a week before the experiment, a standard diet, and water ad libitum. The rats were then randomly divided into 2 groups (*n* = 6 per group): control and medicine groups. The dosage of TCM was converted to human dosage. Therefore, the medicine group was orally administered XKST (0.39 g/kg), and the control group was orally administered an equivalent volume of distilled water. Before the experiment, the rats were allowed for fasting for 12 h and drank water freely.

#### 2.6.2 Collection and Preprocessing of Blood Samples

The blood samples were collected from the fundus venous cluster of rats at 5 min, 15 min, 0.5, 1, 2, 4, 8, 12, and 24 h after administration and then transferred into heparinized tubes. Plasma was separated by centrifugation at 4,000 r/min for 10 min at 4°C, and the supernatant was stored at −80°C for analysis. Plasma (100 μl) was added into methanol (300 μl), vortex-mixed for 30 s, and centrifuged at 13,000 rpm for 10 min to precipitate the proteins. A measure of 300 μl of protein-free supernatant was collected and dried with nitrogen at room temperature. The dried residue was reconstituted in 150 μl of methanol. After centrifugation for 5 min at 13,000 rpm, an aliquot of 2 μl was injected for UPLC/MS analysis.

#### 2.6.3 Data Acquisition

Chromatographic separation was carried out using an Ultimate 3000 UHPLC system (Thermo Fisher Scientific Inc., MA, United States). The mobile phase of (A) 0.05% (by volume) formic acid in water and (B) acetonitrile was accustomed. Other chromatographic conditions were the same as those in “2.3.1.” A hybrid quadrupole-orbit trap mass spectrometry (Thermo LC/MS Division, CA, United States) was used to carry out mass spectrometry with an electrospray ionization source (ESI) operating in a negative ion mode. MS operating conditions were set as follows: ion spray voltage at 3,600 V, capillary temperature at 320°C, aux gas heater temperature at 380°C, sheath gas flow rate at 35 arb, aux gas flow rate at 10 arb, sweep gas flow rate at 2 arb, and spray voltage at 2.8 kV. For full scan MS analysis, the spectra were recorded in the range of m/z100-1300 with a resolution of 70000. In the MS/MS mode, the impact energy was 10 eV.

### 2.7 Quantification of Quality Markers of Sixteen Batches of XKST by HPLC

After quality markers were determined by the earlier research study, the contents of quality markers of 16 batches of XKST were determined using the validated HPLC method under item “2.3.”

### 2.8 Statistical Analysis

The Similarity Evaluation System for Chromatographic Fingerprint of TCM (2004 editions) was used to process HPLC fingerprints. SPSS 16.0 statistical software was used to process statistical data, and SIMCA-P 12.0 (Umetrics) was used for PLSR analysis. Compound Discoverer 3.0 (Thermo Fisher Scientific) was employed to identify the compounds. Pharmacodynamics statistical analyses were performed by GraphPad Prism 6.01 (GraphPad Software Inc., La Jolla, CA, United States). One-way analysis of variance was used to analyze the statistical significance of any difference among groups. The data were expressed as the means ± standard error (SE). Differences were considered significant when p was less than 0.05 or 0.01.

## 3 Results and Discussion

### 3.1 Chromatographic Fingerprint Analysis

#### 3.1.1 Method Validation

In order to validate the applicability of present chromatographic conditions shown in Section 2.3.1, a sample S2 solution (batch no. 0181248) was used for method validation. As shown in Supplementary Table S1, with all fingerprint peaks calculated, the precision of the method was calculated via consecutively injecting sample solution S2 six times, the relative standard deviation (RSD) of retention times (RTs) and peak areas (PAs) were not more 0.72% and 0.51%, respectively. The repeatability test was validated by injecting six parallel sample solutions, the repeatability RSD of RTs and PAs did not exceed 1.47% and 0.66%, respectively. The stability test of sample solutions was investigated by analyzing the S2 solution stored under the room temperature (20 ± °C) at 0, 2, 4, 6, 8, 10, 12, 14, and 16 h, and the RSD of RTs and PAs turned out below 0.66% and 0.60%, respectively. All test results earlier demonstrated that this chromatographic method was reliable in XKST fingerprint analysis.

#### 3.1.2 Fingerprint Analysis of XKST

**FIGURE 2:**
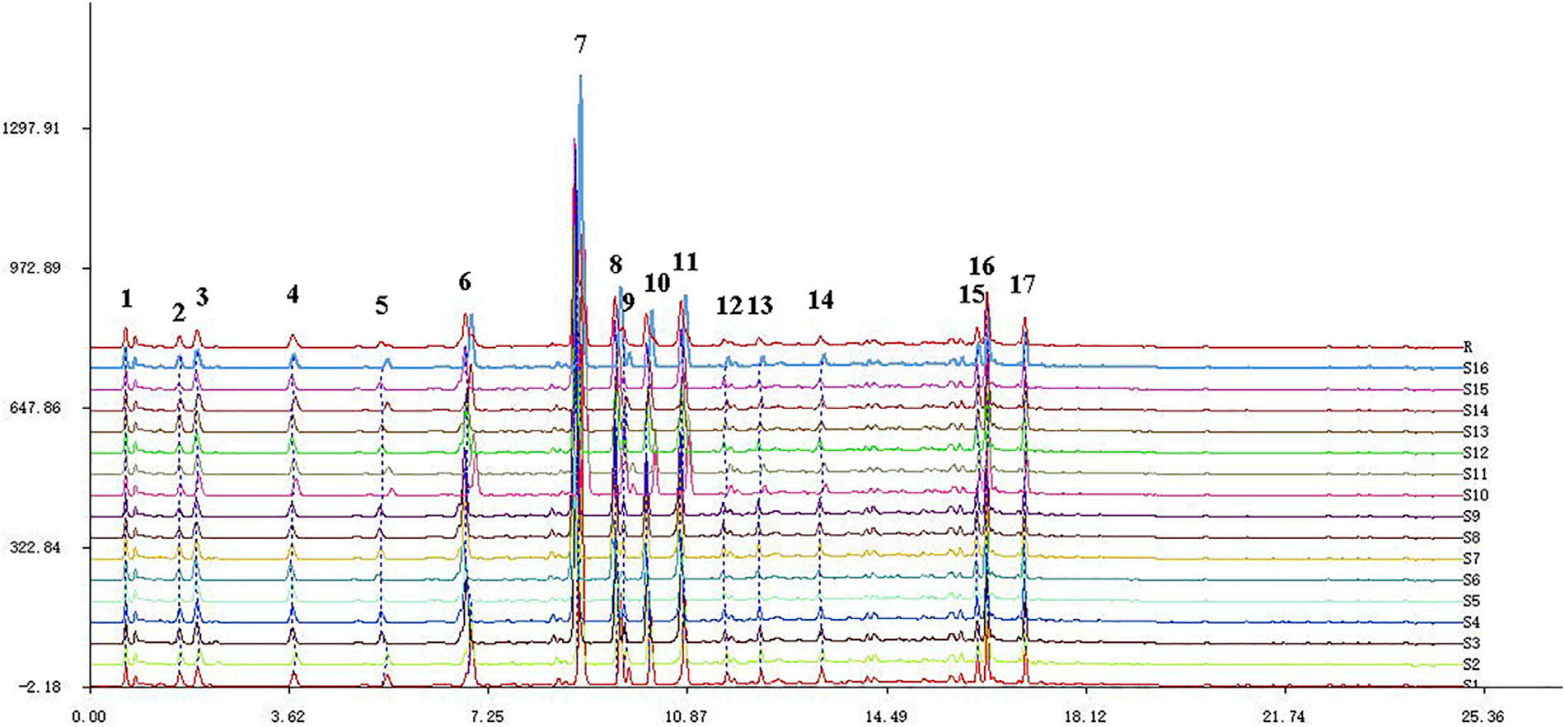
HPLC fingerprints of 16 batches of XKST and reference fingerprint.

The obtained fingerprints were imported into the Similarity Evaluation System for Chromatographic Fingerprint of TCM (2004 editions). After two-point correction, 17 chromatographic common peaks were identified according to the alignment results, and the control fingerprint was generated. The peak area percentages of the 17 chromatographic common peaks were all greater than 1%, and the separation was good, which could be used for quantification. The fingerprints of S1–S16 and control fingerprint (R) are presented in Figure 2. By comparing the chromatographic peaks of the reference substance, chromatographic peaks 3, 4, 6, 7, 8, 11, 15, 16, and 17 were identified as danshensu, protocatechualdehyde, 3′-hydroxypuerarin, puerarin, 3′-methoxypuerarin, daidzin, daidzein, salvianolic acid B, and salvianolic acid A, respectively. Meanwhile, these five ingredients, salvianolic acid D, ononin, quinic acid, puerarin, and biochanin A, were also compared with controls and were not among the 17 chromatographic common peaks due to their low levels in XKST.

### 3.2 Determination of the Antiarrhythmic Effect of XKST

As shown in Table 1, the antiarrhythmic effect of 16 batches of XKST was determined by the heart rate recovery rate of zebrafish larvae. The closer the heart rate was to that of the control group, the stronger was the antiarrhythmic effect. The heart rate per minute of each experimental group was significantly higher than that of the model group, indicating that XKST had a pharmacological effect on the antiarrhythmia of zebrafish. At the same time, there were some differences in heart rate recovery among different batches of samples.

**TABLE 1:** Heart rate recovery rate of zebrafish larvae treated with XKST.

Group	Concentration	Heart rate per minute	Heart rate recovery rate/%
Control	—	162.82 ± 4.67	100
Model	—	98.06 ± 6.55^##^	0
ISO	20 μM	152.73 ± 4.73**	84.42
S1	200 μg/ml	143.77 ± 5.54**	70.58
S2	200 μg/ml	150.64 ± 6.25**	81.2
S3	200 μg/ml	137.54 ± 7.33**	60.96
S4	200 μg/ml	135.75 ± 7.14**	58.2
S5	200 μg/ml	131.00 ± 8.83**	50.86
S6	200 μg/ml	138.75 ± 8.00**	62.83
S7	200 μg/ml	131.31 ± 7.26**	51.34
S8	200 μg/ml	141.21 ± 6.17**	66.64
S9	200 μg/ml	141.69 ± 7.57**	67.38
S10	200 μg/ml	114.55 ± 8.68**	25.45
S11	200 μg/ml	127.33 ± 8.37**	45.2
S12	200 μg/ml	131.45 ± 6.68**	51.57
S13	200 μg/ml	124.50 ± 5.59**	40.83
S14	200 μg/ml	141.82 ± 6.59**	67.57
S15	200 μg/ml	141.00 ± 7.12**	66.31
S16	200 μg/ml	129.00 ± 7.25**	47.78

^#^ *p* < 0.05 vs. control, ^##^ *p* < 0.01 vs. control, *p < 0.05 vs. model, **p < 0.01 vs. model.

### 3.3 Establishment and Verification of the Spectrum-Effect Relationship Model of XKST

To establish the spectrum-effect relationship model of XKST, 16 batches of samples of XKST should be divided into the calibration set and validation set. The Kennard–Stone (KS) method starts by initially identifying two samples with the highest Euclidean distance. Subsequently, the sample with the lowest Euclidean distance from the already selected samples is included in the representative subset. The method is repeated till the required number of samples or objects are included in the training set (Singh et al., 2019). Sample set partitioning based on the joint x–y distance (SPXY) is another sample set partitioning method that takes both x and y variables into account when calculating the distance between samples based on the KS method (He et al., 2015). In order to verify the accuracy of the experimental results, we used these two grouping methods, and 11 batches of samples were selected as a calibration set and the remaining five batches were selected as a validation set for verification. SIMCA-P12.0 processing software was used to analyze the correlation between the peak areas of 17 common peaks of XKST fingerprints and heart rate recovery rate by partial least squares regression (PLSR), and cumulative cross-validated coefficient (Q^2^) was small, indicating that the established model of spectrum-effect relationship was not accurate. This may be attributed to the complexity of the XKST system, which contained more interference information in its fingerprints. In order to filter out information unrelated to the heart rate recovery rate, orthogonal signal correction (OSC) was used in this study. OSC can be utilized to remove systematic variation from the response matrix *X* that is unrelated, or orthogonal, to the property matrix *Y*, so that OSC can simplify the model and improve its prediction ability (Niazi and Goodarzi, 2008). After OSC, the PLSR method was utilized to establish the spectrum-effect relationship between XKST fingerprints and heart rate recovery rate, and the cross validation (CV) method was used to optimize the model parameters. The cumulative multiple correlation coefficient (*R* ^*2*^) represents the degree of fit between the model and the data. Generally, a higher *R* ^*2*^ (close to 1) indicates that a better model is more likely to be obtained. *Q* ^*2*^ represents the prediction performance of the model in cross validation, and the greater the value (>0.5 in general), the better is the prediction performance of the model. As indicated in Table 2, when the data preprocessing method was OSC and the number of principal components was 1, the *Q* ^*2*^ value and the accumulated *R* ^*2*^ *Y* are closer to 1 than untreated, and the parameters of the model are appropriate.

**TABLE 2:** Optimized results of the PLS model.

Grouping method	Pretreatment method	Number of OSC	PLS component number	Cumulative *R* ^*2*^ *X*	Cumulative *R* ^*2*^ *Y*	*Q* ^*2*^
—	PLSR	0	2	0.752	0.654	0.340
KS	OSC–PLSR	1	1	0.873	0.985	0.980
SPXY	OSC–PLSR	1	1	0.889	0.979	0.966

**FIGURE 3:**
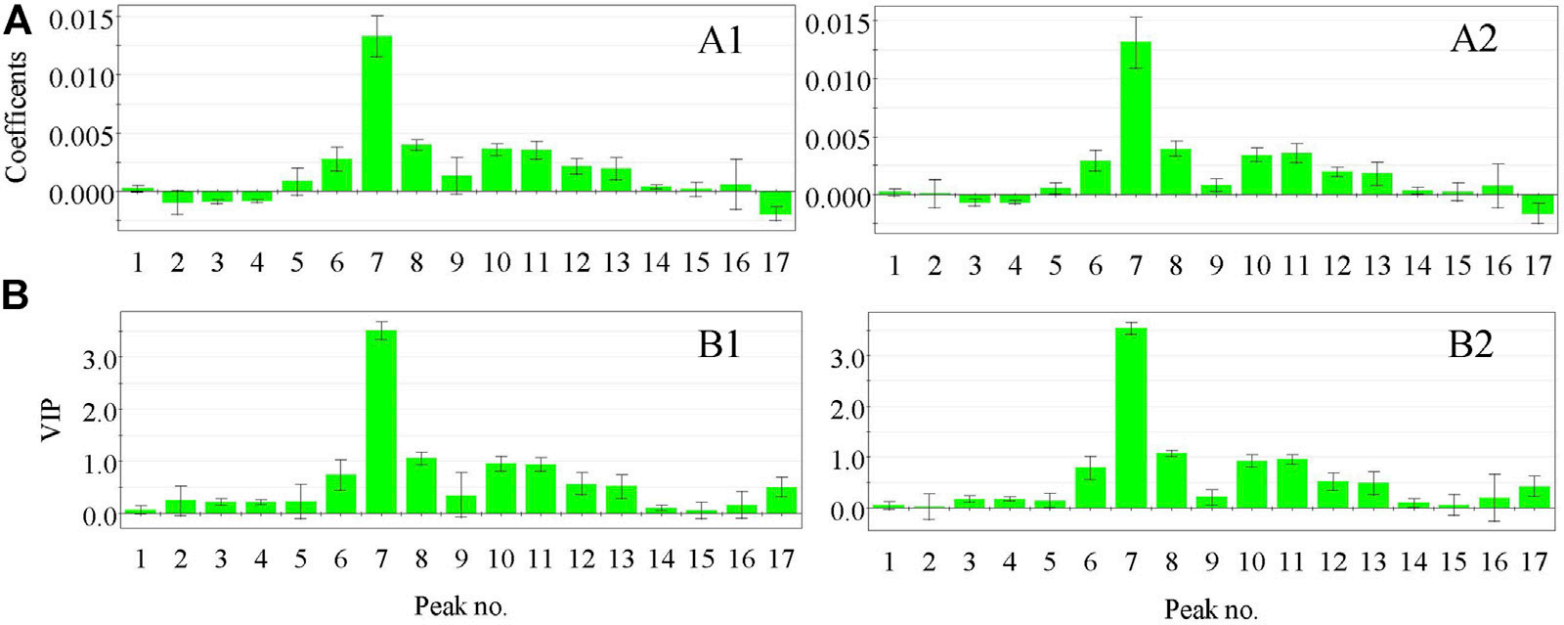
Coefficient plot **(A)** and VIP plot **(B)** of the OSC–PLS model grouped using the KS and SPXY method. **(A1)** Coefficient plot of the OSC–PLS model grouped using the KS method, **(B1)** VIP plot of the OSC–PLS model grouped using the KS method, **(A2)** Coefficient plot of the OSC–PLS model grouped using the SPXY method, and **(B2)** VIP plot of the OSC–PLS model grouped using the SPXY method.

**FIGURE 4:**
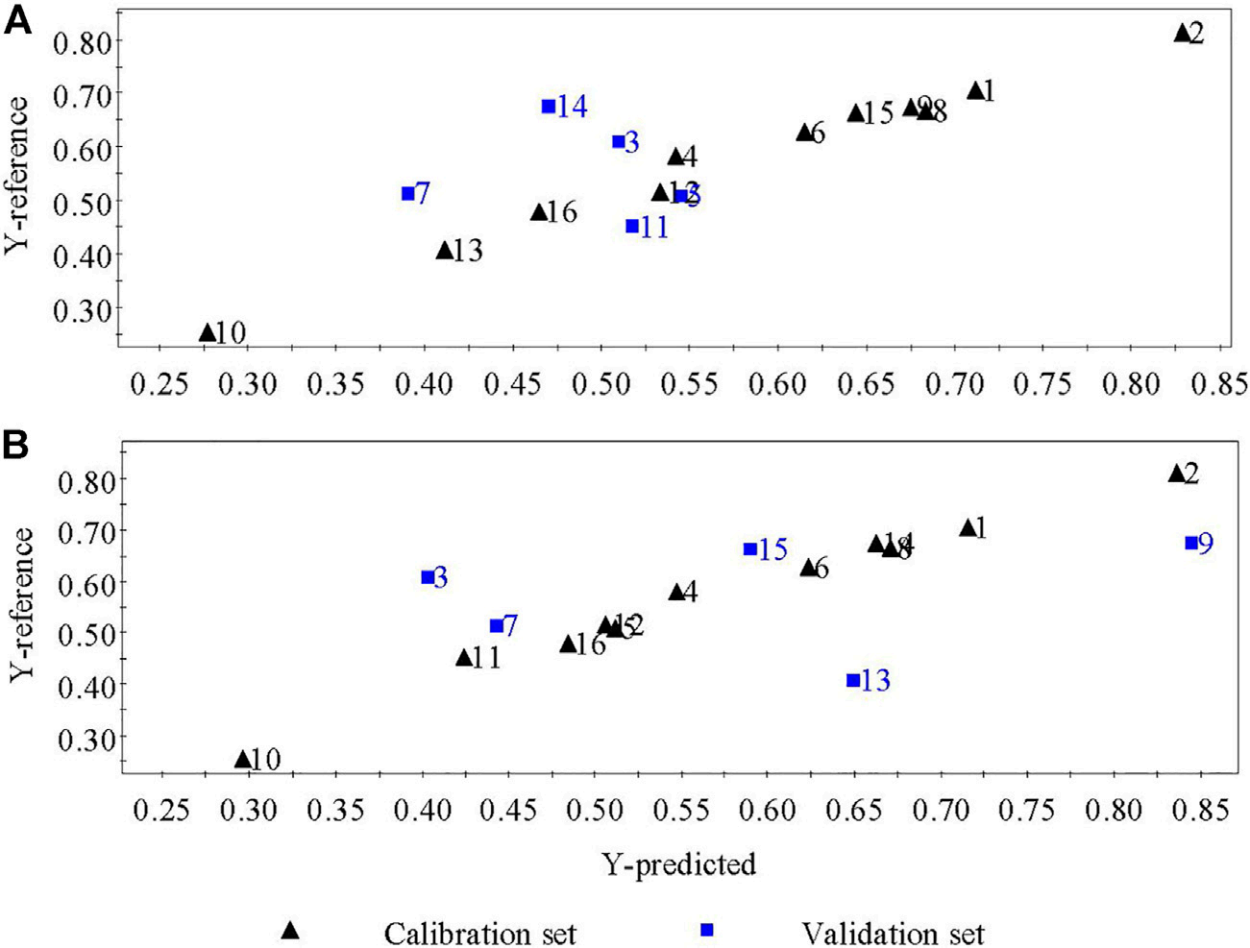
Predicted results of the OSC–PLSR model grouped using the KS **(A)** and SPXY methods **(B)**.

Standardized regression coefficients and the variable importance in projection (VIP) of the common peaks of the fingerprints calculated based on the optimized OSC–PLSR model are shown in Figure 3. As can be seen from Figure 3 that the coefficient plot and VIP plot of OSC–PLS model grouped using the KS and SPXY method were basically similar respectively, which indicated the reliability of the results. In order to ensure the accuracy of the results, we took the same results obtained by the two grouping methods as the evaluation basis. It can be seen from Figures 3A1,A2 that chromatographic peaks No. 1, 5, 6, 7, 8, 9, 10, 11, 12, 13, 14, 15, and 16 in the XKST fingerprints were positively correlated with the heart rate recovery rate, indicating that heart rate recovery rate of the sample would be enhanced with the increase of the peak areas of these chromatographic peaks. Among them, chromatographic peaks No. 6, 7, 8, 9, 10, 11, 12, 13, and 14 had statistical significance due to the confidence interval of not crossing zero in the coefficient plot of the OSC–PLS model, while chromatographic peaks No. 1, 15, and 16 had no statistical significance due to the confidence interval of crossing zero in the coefficient plot of the OSC–PLS model. Moreover, chromatographic peaks No. 3, 4, and 17 were negatively correlated with heart rate recovery rate, all of which were statistically significant. VIP is an indicator used to assess the importance of the independent variable in explaining the dependent variable. The greater the value, the greater the contribution of the variable to the dependent variable. Generally, VIP greater than 1 was regarded as the standard for variable selection, and it was believed that the independent variable, that is, fingerprint chromatographic peak, in which VIP >1 had a significant influence on the dependent variable (heart rate recovery rate) (Afanador et al., 2013). Figures 3B1,B2 showed that the VIP values of chromatographic peaks No. 7 and 8 exceed 1, and these peaks have a significant effect on the heart rate recovery rate. According to the normalized regression coefficient plot and VIP plot, chromatographic peaks No. 7 and 8 were positively correlated with the heart rate recovery rate. The optimized OSC–PLSR model was established to verify the prediction of heart rate recovery rate of the validation sample set, and the results were presented in Figure 4. As can be seen from Figures 4A,B, the model predicted the values of the calibration set samples and the validation set samples (S3, S5, S7, S11, and S14) grouped using KS method have a good correlation with the reference values (*R* ^*2*^ *Y* = 0.7690), and the model predicted values of the calibration set samples and the validation set samples (S3, S7, S9, S13, and S15) grouped using the SPXY method have a good correlation with the reference values (*R* ^*2*^ *Y* = 0.7292) as well, indicating that the established model could predict the heart rate recovery rate through the fingerprints of these samples.

By comparing the HPLC chromatographic peaks of the standard substances and the corresponding samples, the peaks No. 7 and 8 were identified as puerarin and 3′-methoxypuerarin, respectively. Therefore, the antiarrhythmic components of XKST were preliminarily determined as puerarin and 3′-methoxypuerarin by spectrum-effect relationship.

### 3.4 AntiCardiovascular Disease Components Were Screened Based on Network Pharmacology

#### 3.4.1 Determination of Anticardiovascular Disease Components

A total of 519 potential targets of XKST chemical components were predicted and collected through the Swiss Target Prediction Database, PubChem Database, and STITCH Database. A total of 275 targets of CVDs reported were searched through OMIM database, Drugbank database, PharmGKB database and KEGG database. After the integration of the two types of targets, 62 mapping targets were obtained, which were regarded as the predicted targets of XKST in the treatment of CVDs. Through Cytoscape analysis, a total of 44 anticardiovascular disease components related to the predicted targets were identified, as shown in Table 3.

**TABLE 3:** Forty-four anticardiovascular disease components of XKST identified by network pharmacology.

Serial number	Compound name	Serial number	Compound name
XKST-01	Quinic acid	XKST-23	Ginsenoside Rb1
XKST-02	Malic acid	XKST-24	Ginsenoside Rg2
XKST-03	Danshensu	XKST-25	Ginsenoside Rh1
XKST-04	Chlorogenic acid	XKST-26	Formononetin
XKST-05	Puerarin	XKST-27	Ginsenoside Rd
XKST-06	Daidzin	XKST-28	Notoginsenoside K
XKST-07	Genistein-8-C-glucoside	XKST-29	Ginsenoside Rg3
XKST-08	Hyperoside	XKST-30	Salvianolic acid F
XKST-09	Apigenin-7-O-glucoside	XKST-31	Salvianolic acid G
XKST-10	Salvianolic acid D	XKST-32	Protocatechuic acid
XKST-11	Rosmarinic acid	XKST-33	Sophoraside A
XKST-12	Ononin	XKST-34	Caffeic Acid
XKST-13	Notoginsenoside R1	XKST-35	Genistein-8-C-glucoside
XKST-14	Daidzein	XKST-36	Lithospermic acid
XKST-15	Ginsenoside Rg1	XKST-37	Pueroside B
XKST-16	Ginsenoside Re	XKST-38	4′,6-Dimethoxyisoflavone-7-O-glucoside
XKST-17	Biochanin A	XKST-39	5′-Hydroxyl oninin
XKST-18	Salvianolic acid A	XKST-40	Daidzein-4′,7-diglucoside
XKST-19	Salvianolic acid B	XKST-41	Cumoesterol
XKST-20	Genistein	XKST-42	Costunolide
XKST-21	Salvianolic acid C	XKST-43	Dehydrocostus lactone
XKST-22	Notoginsenoside R2	XKST-44	Oleanic acid

#### 3.4.2 Compound–Target Interaction Network of XKST

**FIGURE 5:**
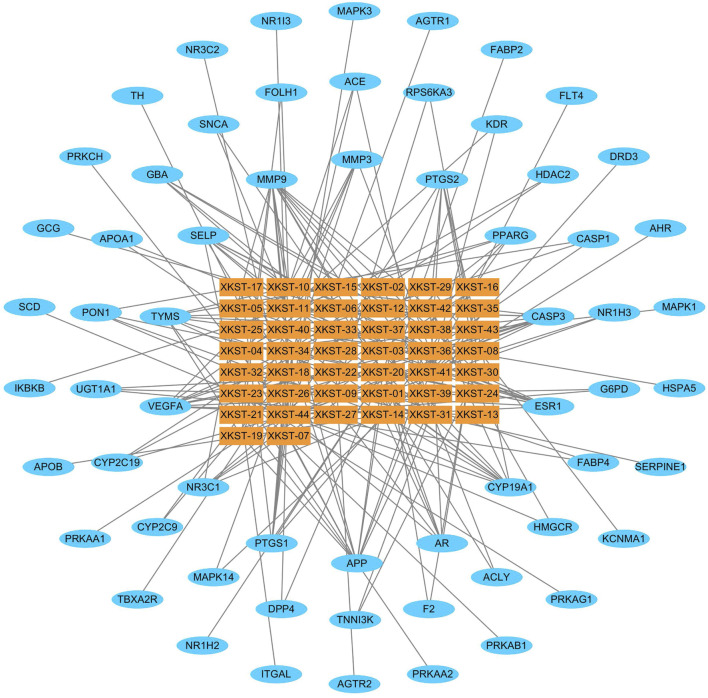
Compound–target network of XKST.

The Cytoscape 3.2.1 software was used to establish a compound–target interaction network with 44 compounds and their mapping targets as shown in Figure 5. The compound–target network consisted of 44 compound nodes and 62 target nodes. The rectangular nodes represent the compounds, and the elliptical nodes represent the active targets corresponding to the compounds. The nodes were distributed from inside to outside according to the degree of freedom, and each edge represents the interactive relationship between the compounds and the targets. There were 15 compounds with more than 5 targets of actions. Among 62 potential targets, 16 targets were linked to more than three compounds, reflecting the mechanism of multi-component and multi-target interaction of XKST, which are in line with the action characteristics of TCM.

#### 3.4.3 KEGG Pathway Enrichment Analysis

**FIGURE 6:**
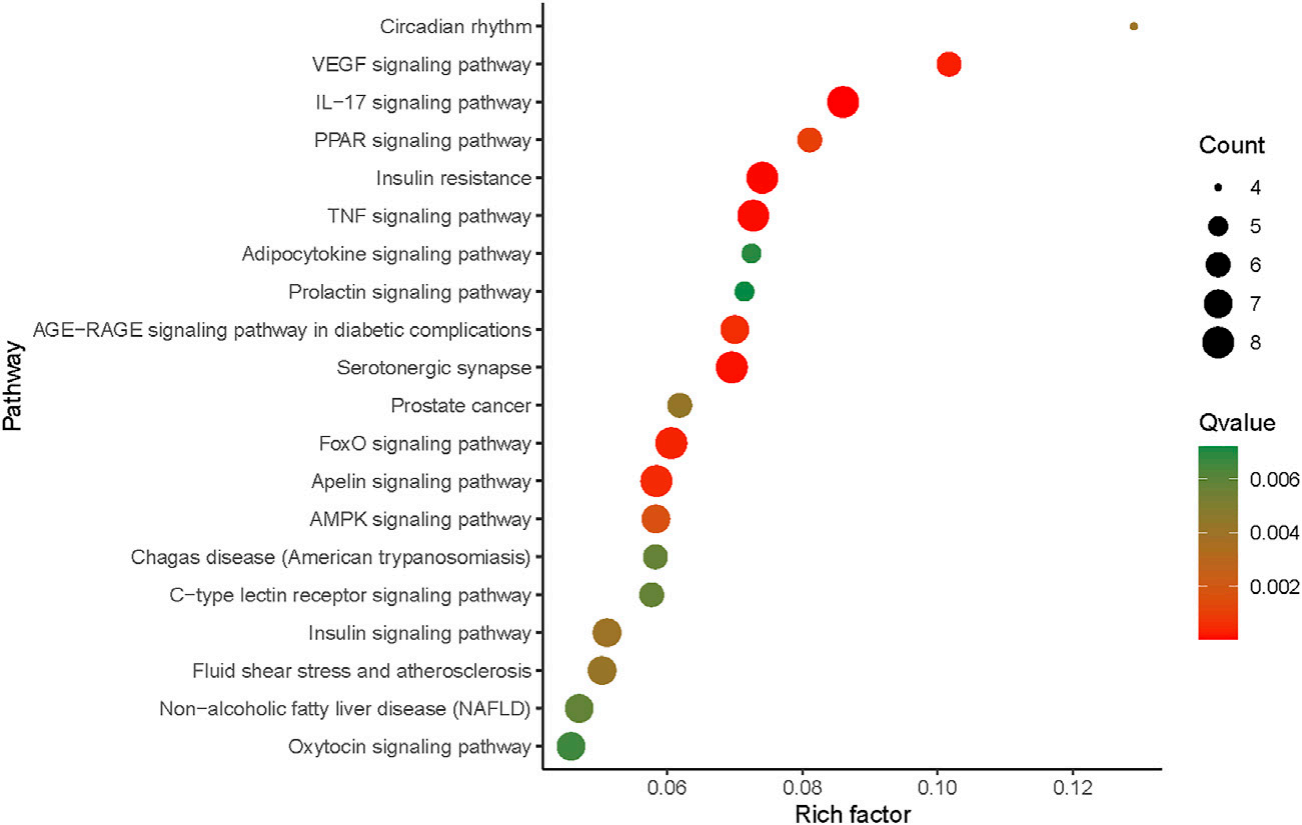
KEGG mechanism analysis.

Sixty-two target proteins involved in the XKST compound–target network were enriched by the KEGG pathway analysis, and 34 action pathways of XKST in the treatment of cardiovascular diseases were screened with *p* value <0.05, suggesting that the potential active components of XKST may treat CVDs by acting on these signaling pathways. After sorting by the Q value, the information of the top 20 pathways with a smaller Q value was imported into R software to obtain KEGG mechanism analysis results (Figure 6). The abscissa was a rich factor, which was the ratio of the number of differential genes under this metabolic pathway to the number of genes annotated to this pathway. The larger the value was, the greater the enrichment degree was. The bubble size in the figure indicates the number of enrichment genes in this pathway. The larger the bubble, the more the number of enrichment genes. The color difference of bubbles indicated the enrichment degree of targets in this pathway, and the redder the color was, the higher the enrichment degree was. The pathways with a higher enrichment degree and more enrichment number of targets were the VEGF signaling pathway and IL-17 signaling pathway. Several studies have reported that VEGF is an important vascular growth factor for both the development and maintenance of the vascular system, and systemic VEGF inhibition disrupts endothelial homeostasis and accelerates atherogenesis, suggesting that VEGF resistance plays a great role in CVDs (Schmidt, 2012; Winnik et al., 2013). CVDs are characterized by obstructive blood flow and chronic inflammatory diseases, in which the circulating IL-17 and other inflammatory factors act on blood vessels, immune cells, and cardiac cells contributing directly or indirectly to chronic inflammation, cell apoptosis, coagulation, and thrombosis and leading eventually to destabilization and plaque rupture. (Ding et al., 2012; Phoksawat et al., 2020). All these studies support the accuracy of the target pathway we have found.

### 3.5 UHPLC/MS–MS Analysis of Constituents of XKST Dissolved in Plasma

**FIGURE 7:**
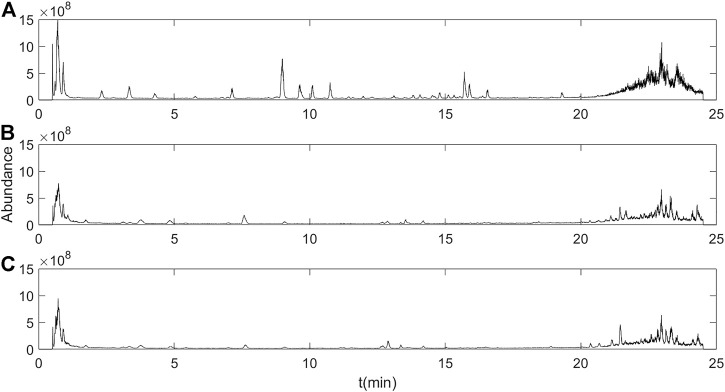
Total ion chromatogram of XKST **(A)**, blank plasma **(B)**, and plasma-containing drug **(C)**.

The XKST mass spectrometry information was collected, and plasma samples at nine blood collection time points were analyzed. It was found that the plasma samples at 1 h after gavage administration of XKST had the highest ion strength in the total ion chromatogram. Meanwhile, as shown in Supplementary Figure S1, according to plasma peak area versus time profiles for constituents after the oral administration of XKST in male rats, the peak time of most components was 1 h, or they were not completely metabolized at 1 h, which can be detected. It has been reported that most drug concentrations in the blood of rats can reach a peak within 1–2 h after taking TCM, which is consistent with the physiological characteristics of drug absorption into the blood of rats, and the plasma obtained at this time is drug-containing plasma. (Mehta et al., 2015; Hanafy et al., 2021). Therefore, in this study, the plasma samples at 1 h after administration were used for the analysis of components in plasma. By comparing the total ion chromatogram of XKST administration rat plasma and blank plasma samples (Figure 7), the components of plasma collected at 1 h after oral administration of XKST were extracted with greater significance than those of the blank plasma (*p* < 0.05) in the UHPLC/MS–MS data, and these components were seen as compounds of XKST absorbed and exposed *in vivo*, as illustrated in Table 4. A total of 20 compounds, including four phenolic acids, two esterosides, one hydroxy acid, one isoflavones, and twelve isoflavone glycosides, were identified in plasma samples using literature information and Compound Discoverer 3.0, which was used to match the mass spectrum information. These components in plasma may be the potentially active components of XKST and be directly related to the pharmacological effects.

**TABLE 4:** Identification of compounds of XKST absorbed and exposed *in vivo*

Number	t R/min	Compound	Formula	Parent ion (*m/z*)	Theoretical value (*m/z*)	Error ( × 10^−6^)	Daughter ion (m/z)
1	2.39	Danshensu	C_9_H_10_O_5_	197.0446 [M−H]^−^	197.0448	−1.6544	179 [M−H−H_2_O]^−^
2	5.75	Puerarin-7-O-glucoside	C_27_H_30_O_14_	577.1667 [M−H]^−^	577.1556	19.1040	457 [M−H−C_4_H_8_O_4_]^−^
3	6.98	Daidzin-4′-O-glucoside	C_27_H_30_O_14_	623.1628 [M + HCOO]^−^	623.1612	2.6446	415 [M−H−C_6_H_10_O_5_]^−^
4	7.15	3′- Hydroxypuerarin	C_21_H_20_O_10_	431.0985 [M−H]^−^	431.0978	1.7351	311 [M−H−C_4_H_8_O_4_]^−^
5	9.01	Puerarin	C_21_H_20_O_9_	415.1035 [M−H]^−^	415.1028	1.6430	295 [M−H−C_4_H_8_O_4_]^−^
6	9.67	Quinic acid	C_7_H_12_O_6_	191.0052 [M−H]^−^	191.0555	−4.6322	147 [M−H−CO_2_]^−^
							111 [M−H−CO_2_−2H_2_O]^−^
7	10.14	Mirificin	C_26_H_28_O_13_	547.1466 [M−H]^−^	547.1450	2.7141	295 [M−H−C_5_H_8_O_4_−C_4_H_8_O_4_]^−^
8	10.62	3′-Methoxy puerarin 6'' -O-ß-Apionoside	C_27_H_30_O_14_	577.1567 [M−H]^−^	577.1556	1.7777	325 [M−H−C_5_H_8_O_4_−C_4_H_8_O_4_]−
9	11.41	3′-Methoxy daidzin	C_22_H_22_O_10_	491.1204 [M + HCOO]^−^	491.1189	3.0848	445 [M−H]^−^
							283 [M−H−C6H10O5]^−^
10	12.04	Genistein-8-C-apiosyl (1-6)-glucoside	C_26_H_28_O_14_	563.1420 [M−H]^−^	563.1399	3.5728	311 [M−H−C_5_H_8_O_4_−C_4_H_8_O_4_]^−^
11	12.3	Pueroside A	C_29_H_34_O_14_	605.1890 [M−H]^−^	605.1869	3.3295	297 [M−H−C_6_H_10_O_4_−C_6_H_10_O_5_]^−^
12	13.76	4′- Methoxypuerarin	C_22_H_22_O_9_	429.1187 [M−H]^−^	429.1185	0.5290	309 [M−H−C_4_H_8_O_4_]^−^
13	13.94	Formononetin-8-C	C_27_H_30_O_13_	561.1623 [M−H]^−^	561.1606	2.7800	309 [M−H−C_5_H_8_O_4_−C_4_H_8_O_4_]^−^
		-glucoside-O-xyloside					
14	14.02	Salvianolic acid D	C_20_H_18_O_10_	417.0836 [M−H]^−^	417.0820	3.6060	373 [M−H−CO_2_]^−^
							175 [M−H−CO_2_−C_9_H_10_O_5_]^−^
15	15.29	Sophoraside A	C_24_H_26_O_10_	519.1519 [M + HCOO]^−^	519.1502	3.3401	311 [M−H−C_6_H_10_O_5_]^−^
							267 [M−H−C_6_H_10_O_5_−CO_2_]^−^
16	15.65	Ononin	C_22_H_22_O_9_	475.1252 [M + HCOO]^−^	475.1240	2.6288	267 [M−H−C_6_H_10_O_5_]^−^
17	15.9	Daidzein	C_15_H_10_O_4_	253.0506 [M−H]^−^	253.0500	2.3434	224 [M−H−CHO]^−^
							209 [M−H−CO_2_]^−^
18	16.3	Biochanin A	C_16_H_12_O_5_	283.0617 [M−H]^−^	283.0606	3.9532	268 [M−H−CH_3_]^−^
19	16.5	Salvianolic acid A	C_26_H_22_O_10_	493.1153 [M−H]^−^	493.2588	3.8389	295 [M−H−C_9_H_10_O_5_]^−^
20	16.74	Salvianolic acid B	C_36_H_30_O_16_	717.1475 [M−H]^−^	717.1454	2.8153	519 [M−H−C_9_H_10_O_5_]^−^

### 3.6 Integrated Analysis

By establishing a model of the spectrum-effect relationship between XKST fingerprints and the heart rate recovery rate, it was preliminarily confirmed that two antiarrhythmic components, namely, puerarin and 3′-methoxypuerarin, have a significant positive correlation with the heart rate recovery rate. The results of the network pharmacological study identified thirty-one potential active components of XKST for the treatment of CVDs. By integrating the two types of experiments, a total of 32 effective components were identified. On the other hand, with plasma pharmacochemistry study, 20 active components were identified from the perspective of drug absorbed and exposed *in vivo*. Nine components, danshensu, salvianolic acid A, salvianolic acid B, salvianolic acid D, ononin, quinic acid, puerarin, daidzein, and biochanin A, were obtained by the intersection of active components absorbed and exposed *in vivo* and the aforementioned 32 effective components. These nine components not only satisfied the activity of treating CVDs, but also satisfied the absorption of prototype components by the body. In the five determination principles of quality markers, these nine active components conform to delivery and traceability, specificity and effectiveness. However, salvianolic acid D, ononin, quinic acid, puerarin, and biochanin A violated the principle of testability because they are too small to be measured in the drug as has been tested in this study. Among the remaining ingredients, danshensu, salvianolic acid B, and salvianolic acid A are derived from danshen (Mei et al., 2021), which is the king (Jun) medicine of XKST, while puerarin and daidzein are derived from Gegen (Niu et al., 2012), which is the minister (Chen) medicine of XKST. The composition of these five components conforms to the principle of compatibility. In summary, the potential quality markers of XKST were preliminarily identified as danshensu, salvianolic acid B, salvianolic acid A, puerarin, and daidzein. Studies have shown that danshensu, salvianolic acid B, and puerarin have the activity of inhibiting free radical production and antioxidants and play a definite role in the treatment of CVDs (Lam et al., 2007; Wu et al., 2008; Zhao et al., 2008; Chen et al., 2011; Xue et al., 2014; Li et al., 2020; Wang et al., 2020). Meanwhile, danshensu, salvianolic acid B, salvianolic acid A, puerarin, and daidzein all have been noted that they can reduce myocardial injury in a rat ischemia/reperfusion model (Kim et al., 2009; Yin et al., 2013; Xue et al., 2014; Wang et al., 2020; Ling et al., 2021). The aforementioned results confirmed the validity of quality markers preliminarily determined. These components were identified as the potential quality markers of XKST and all have the pharmacological activity of prevention or treatment of CVDs, and are inherent components of XKST that could be measured.

### 3.7 Validation of Quality Markers With Pharmacodynamics of Zebrafish

**FIGURE 8:**
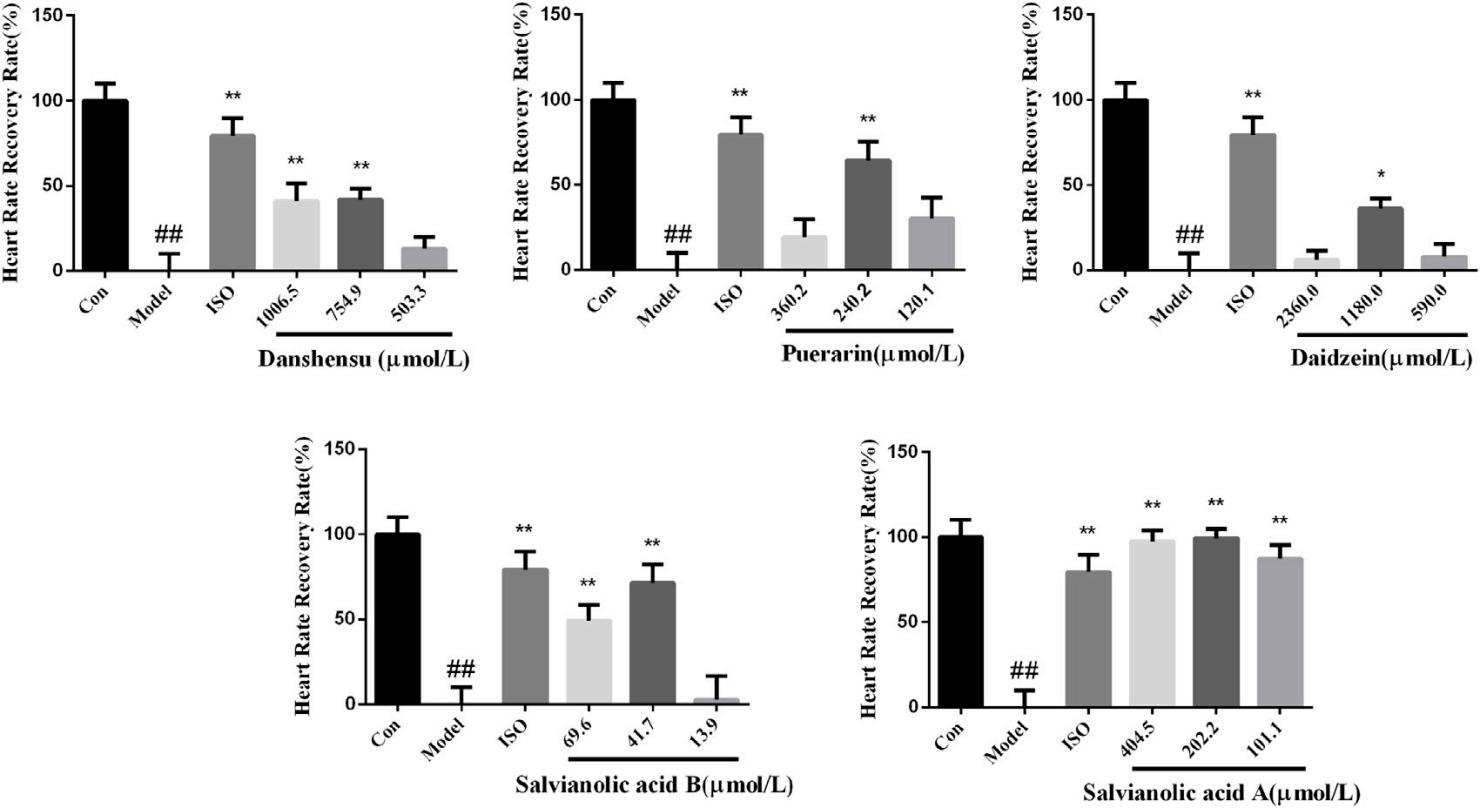
Heart rate recovery rate of zebrafish larvae treated with compounds. *n* = 12. ^#^ *p* < 0.05 vs. control, ^##^ *p* < 0.01 vs. control, *p < 0.05 vs. model, and **p < 0.01 vs. model

According to the results of integrated analysis, danshensu, salvianolic acid B, salvianolic acid A, puerarin, and daidzein were selected to validate activity, considering the availability. After pretreated with compounds and 7.5 µM terfenadine for 24 h, the heartbeat of larvae was recorded under a stereomicroscope. The result showed the heart rate of larvae was recovered to varying degrees (Figure 8). The heart rate recovery rate of zebrafish larvae treated with five compounds in a medium dose group was significantly different from that of the model group. Salvianolic acid A exhibited better activities. The five compounds, danshensu, salvianolic acid B, salvianolic acid A, puerarin and daidzein, we found were considered the most important components responsible for the antiarrhythmic effect, and could be quality markers of XKST. Definitely, the pharmacological effect study on the prevention and treatment of zebrafish arrhythmia was only the preliminary validation of quality markers of XKST in the treatment of CVDs, and the pharmacological effects of other CVDs, for example, coronary heart disease angina pectoris, hyperlipidemia, and hypertension, remained to be further verified.

### 3.8 Quantification of the Quality Markers of XKST

#### 3.8.1 Method Validation

As quality markers in XKST, danshensu, salvianolic acid B, salvianolic acid A, puerarin, and daidzein need to be quantified to fulfill the quality evaluation and control of XKST. For the simultaneous determination of five components in XKST from different batches, the proposed HPLC method was applied by comparing the retention times and on-line UV spectra with those of standards. Calibration curves of those five components are determined by a series of mixed reference solution standard (RSS) solution diluents, as shown in Supplementary Table S2. The limit of detection (LOD) and the limit of quantification (LOQ) were separately determined at the standard of S/N = 3.3 and S/N = 10, and the results of LOD and LOQ for each compound are shown in Supplementary Table S2. Three different quantities (low, medium, and high) of the authentic standards were added to the sample S2 (batch no. 0181248) to evaluate the accuracy of the developed analytical method. The mixtures were extracted and quantified as described before. Then, the quantity of each component was subsequently calculated from the corresponding calibration curves. The results are summarized in Supplementary Table S2. The method had a satisfactory accuracy with the overall recovery from 95.31% to 103.61% for five analytes.

#### 3.8.2 Content Determination of Quality Markers of XKST

The contents of danshensu, salvianolic acid B, salvianolic acid A, puerarin, and daidzein were obtained using the calibration curve method. Each sample was determined in triplicate. The results summarized in Table 5 revealed that the RSD values of the contents of five analytes among different batches were very high (6.87%–16.44%, Table 5), and the contents were obviously different among batches. Presumably, the contents may be affected by the instability of the preparation and diverse sources of herbal medicines. It is necessary to further control the quality of XKST, according to the quality markers.(Dong et al., 2019).

**TABLE 5:** Contents (μg/g) of quality markers of XKST.

Sample	Content (μg/g)				
	Danshensu	Puerarin	Daidzein	Salvianolic acid B	Salvianolic acid A
S1	4223.32	28213.79	1035.98	10842.07	2602.99
S2	4645.43	25973.15	755.31	13433.78	2720.34
S3	4548.31	27011.45	850.48	12978.71	3418.48
S4	4346.63	27310.96	853.23	12193.43	3218.52
S5	4834.66	17357.93	687.33	11219.66	3150.94
S6	4368.05	17249.23	716.81	11894.80	2663.78
S7	4376.49	25873.43	836.48	10747.91	3245.27
S8	3457.47	26854.15	990.81	12068.66	2389.18
S9	3666.63	26107.29	897.71	10429.67	2394.46
S10	4926.06	19833.88	791.76	11696.36	2863.85
S11	4502.94	19999.11	805.41	12098.40	2569.36
S12	4369.58	21819.44	909.39	11414.44	2752.03
S13	5075.79	19329.50	787.09	11406.17	2901.71
S14	4080.40	21690.52	955.59	11615.34	2521.53
S15	3751.31	20709.28	938.12	11375.23	2447.42
S16	4034.66	24343.50	931.46	11310.97	2976.10
Average	4325.48	23104.79	858.93	11670.35	2802.25
RSD (%)	10.42	16.23	11.53	6.68	11.58

## 4 Conclusion

In summary, we have presented an integrated strategy to identify and quantify quality markers in XKST based on the spectrum-effect relationship, network pharmacology, plasma pharmacochemistry, and pharmacodynamics of zebrafish. In this research, the intrinsic relationship between quality control components and pharmacological activity could be truly revealed, and quality markers in XKST were quantified so that the quality of XKST could be revealed and controlled comprehensively. This study provided an experimental basis to achieve a comprehensive and reliable quality control evaluation of XKST and also presented a novel and practical research strategy for exploring the quality markers of CPM.

## Data Availability

The data analyzed in this study is subjected to the following licenses/restrictions. The data were commissioned by Shandong Wohua Pharmaceutical Technology Co., Ltd. and cannot be made public without the company’s permission. Requests to access these datasets should be directed to YW, xiyumenghui108@163.com.

## Supplementary Material

Supplementary Figure S1 Plasma peak area versus time profiles for constituents after the oral administration of XKST in male rats. The order of peak appearance is based on the sum of plasma peak area at each time point. Each point represents an average of six determinations, and the error bars are the standard error of measurement (*n* = 6).

Supplementary Table S1 Precisions, stability, repeatability and recovery rate of five analytes.

Supplementary Table S2 Calibration curves, LOD, LOQ, and recovery rate for five analytes analyzed with the optimized HPLC method.
