# Supplementary material for: An Integrated Strategy to Identify and Quantify the Quality Markers of Xinkeshu Tablets Based on Spectrum-Effect Relationship, Network Pharmacology, Plasma Pharmacochemistry, and Pharmacodynamics of Zebrafish
Source: Front Pharmacol. 2022 May 23;13:899038. doi: 10.3389/fphar.2022.899038 (PMC9170229; doi:10.3389/fphar.2022.899038)
Supplement: Supplementary file 1 [file Table1.docx]

**TABLE S1** Precisions, stability, repeatability and recovery rate of five analytes

| Component | Precision (n = 6) | | | Stability (n = 9) | | | Repeatability (n = 6) | |
| --- | --- | --- | --- | --- | --- | --- | --- | --- |
|  | RSD of retention times (%) | RSD of peak areas (%) | RSD of retention times (%) | | RSD of peak areas (%) | RSD of retention times (%) | | RSD of peak areas (%) |
| Danshensu | 0.72 | 0.51 | 0.46 | | 0.25 | 1.47 | | 0.53 |
| Puerarin | 0.43 | 0.11 | 0.66 | | 0.51 | 0.92 | | 0.58 |
| Daidzein | 0.11 | 0.41 | 0.11 | | 0.60 | 0.22 | | 0.66 |
| Salvianolic acid B | 0.11 | 0.33 | 0.10 | | 0.49 | 0.19 | | 0.53 |
| Salvianolic acid A | 0.10 | 0.33 | 0.11 | | 0.42 | 0.18 | | 0.51 |
