# Supplementary material for: An Integrated Strategy to Identify and Quantify the Quality Markers of Xinkeshu Tablets Based on Spectrum-Effect Relationship, Network Pharmacology, Plasma Pharmacochemistry, and Pharmacodynamics of Zebrafish
Source: Front Pharmacol. 2022 May 23;13:899038. doi: 10.3389/fphar.2022.899038 (PMC9170229; doi:10.3389/fphar.2022.899038)
Supplement: Supplementary file 3 [file Table2.docx]

**TABLE S2** Calibration curves, LOD, LOQ and recovery rate for five analytes analyzed with the optimized HPLC method

| Analyte | Calibration curve | Correlation coefficient (*r^2^*) | Linear range (μg/mL) | LOD (μg/mL) | LOD (μg/mL) | | Recovery rate (n = 9) | |
| --- | --- | --- | --- | --- | --- | --- | --- | --- |
|  |  |  |  |  |  |  | Recovery rate (%) | RSD  (%) |
| Danshensu | y = 3.0756x - 5.1717 | 0.9998 | 15.48-154.8 | 0.8457 | 2.5629 | 103.61 | | 2.39 |
| Puerarin | y = 6.7962x + 3.9891 | 1.0000 | 90.06-900.6 | 1.0358 | 3.1388 | 97.68 | | 1.80 |
| Daidzein | y = 11.413x + 2.0548 | 0.9999 | 3.1-31 | 0.0397 | 0.1203 | 96.71 | | 2.06 |
| Salvianolic acid B | y = 2.4229x - 1.5001 | 1.0000 | 50.2-502 | 0.8365 | 2.5348 | 95.31 | | 3.72 |
| Salvianolic acid A | y = 6.0716x - 7.9364 | 0.9996 | 10.18-101.8 | 0.4164 | 1.262 | 103.03 | | 2.33 |
